# Pediatric femoral shaft fracture requiring revision surgery for nonunion associated with vitamin D and K deficiencies: a case report

**DOI:** 10.1186/s13256-023-04325-x

**Published:** 2024-01-18

**Authors:** Shun Igarashi, Koji Nozaka, Tsuyoshi Shirahata, Hiroaki Kijima, Hidetomo Saito, Kimio Saito, Tetsuya Kawano, Naohisa Miyakoshi

**Affiliations:** Department of Orthopedic Surgery, Akita University Graduate School of Medicine, 1-1-1 Hondo, Akita, Akita 010-8543 Japan

**Keywords:** Pediatric femoral shaft fracture, Nonunion, Vitamin deficiency, Titanium elastic nail, Rigid antegrade intramedullary nail

## Abstract

**Background:**

Nonunion of femoral shaft fractures in children is rare, and there is no clear treatment protocol. In this case report, a pediatric femoral shaft fracture that developed in nonunion due to vitamin deficiency after osteosynthesis, which was successfully treated with vitamin augmentation and replacement with a rigid antegrade intramedullary nail, is described.

**Case presentation:**

The patient is an 11-year-old Japanese girl. She injured her right femoral shaft fracture when she hit a wall after kickboarding down a hill and underwent osteosynthesis with a titanium elastic nail. Six months postoperatively, she developed nonunion, was found to be deficient in vitamins D and K, and was started on vitamin supplementation. She underwent replacement with a rigid antegrade intramedullary nail at 7 months postoperatively, and bone union was achieved 3 months after reoperation.

**Conclusion:**

When delayed union of a fracture is observed postoperatively, even in children without underlying disease, the cause of the problem must be investigated and treated promptly.

## Background

Pediatric femoral shaft fractures are relatively rare, accounting for 1.4–1.7% of all pediatric fractures. Because of their strong callus formation, rapid bone healing, and good autocorrection ability, conservative treatment with traction and casting used to be the mainstay of treatment. In recent years, surgical treatment such as external fixation, Ender nails, and elastic intramedullary nails are often chosen, especially in older children, to get them off the bed early and shorten their hospital stay [1]. In particular, elastic intramedullary nails have been reported to be effective for pediatric femoral shaft fractures [2].

In nonunion, it is generally necessary to accurately evaluate the factors and select the appropriate treatment. If nonunion occurs, it can lead to functional disability, multiple surgeries, social and mental effects on the patient and family, and increased medical costs [3]. However, because nonunion is extremely rare in pediatric femoral diaphyseal fractures, there are no clearly established protocols for treatment or management, which must be handled on a case-by-case basis [4]. A case of a pediatric femoral shaft fracture with nonunion due to vitamin D and K deficiencies after osteosynthesis with a titanium elastic nail, which was successfully treated with vitamin supplementation and revision surgery with a rigid antegrade intramedullary nail, is reported.

## Case presentation

**Fig. 1:**
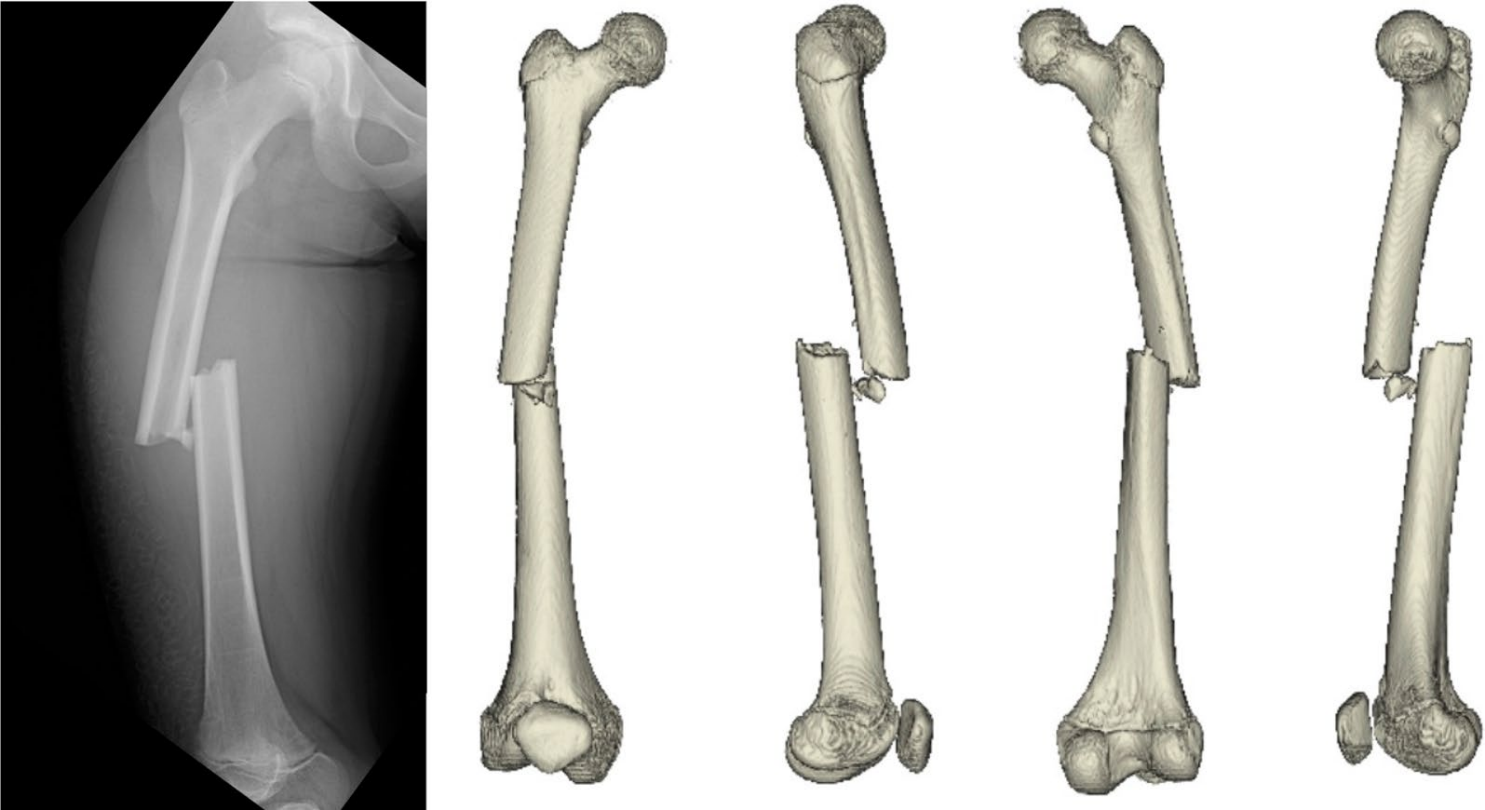
X-ray and computed tomography images of the right femur on admission to the emergency room. Transverse fracture of the right femur with shortening displacement in the middle of the diaphysis is seen

**Fig. 2:**
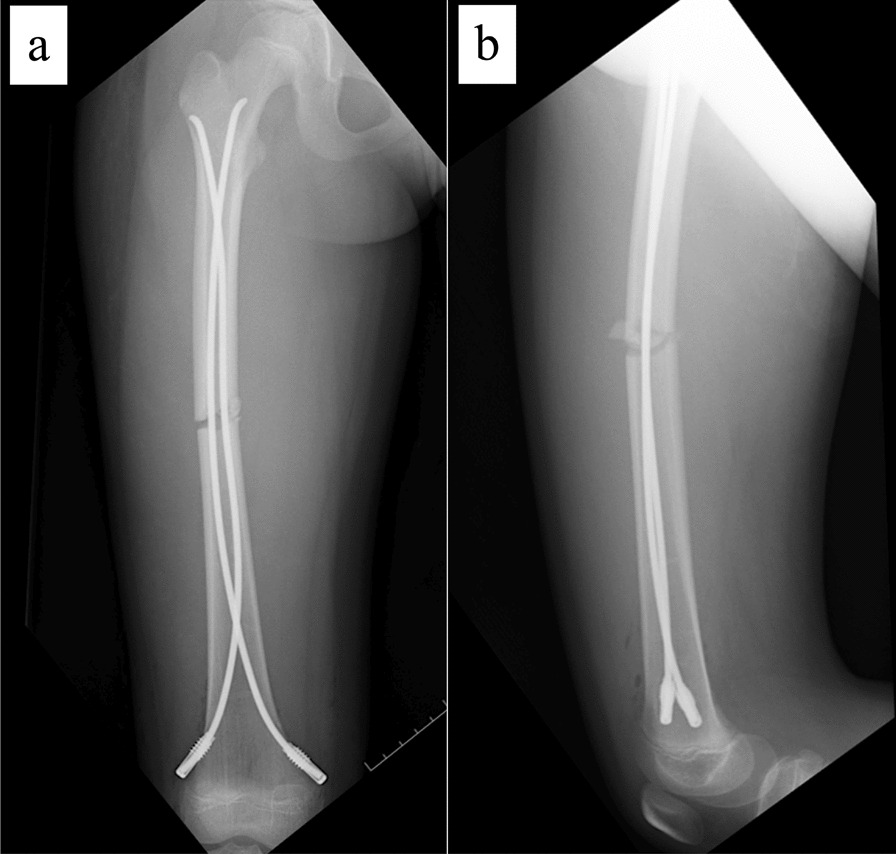
Postoperative radiographs of first surgery. **a** Anteroposterior image and **b** lateral image. Osteosynthesis was performed using two 3.5-mm-diameter nails inserted retrograde

**Fig. 3:**
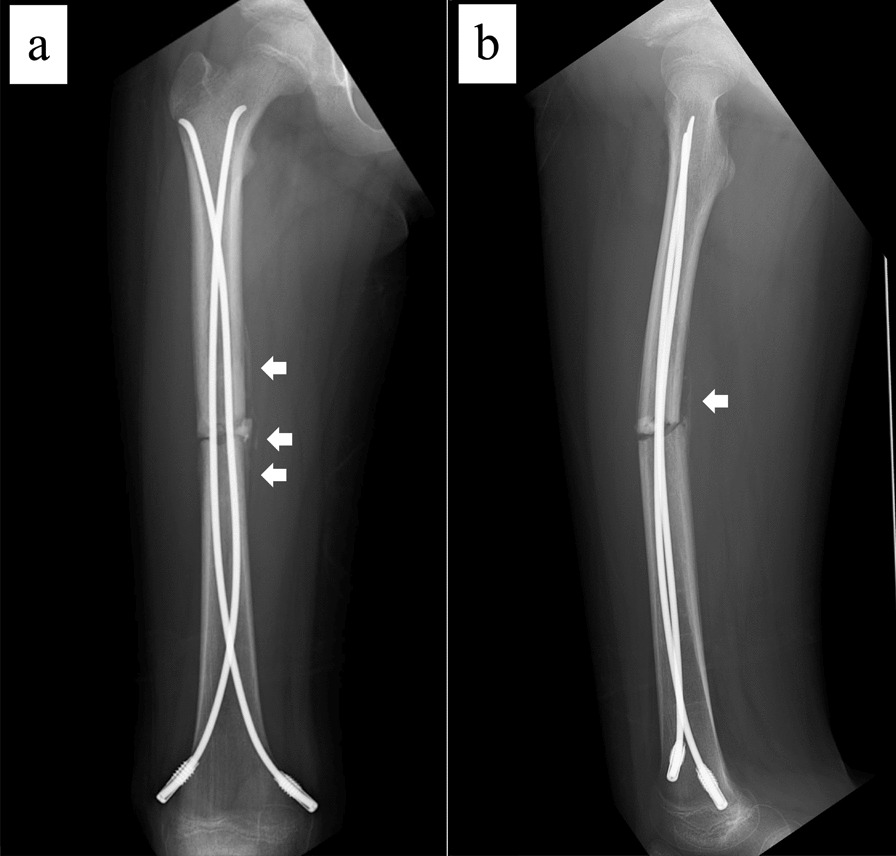
X-ray images at 5 weeks postoperatively. **a** Anteroposterior image and **b** lateral image. Callus formation is observed on the medial and posterior sides of the fracture site (white arrows)

**Fig. 4:**
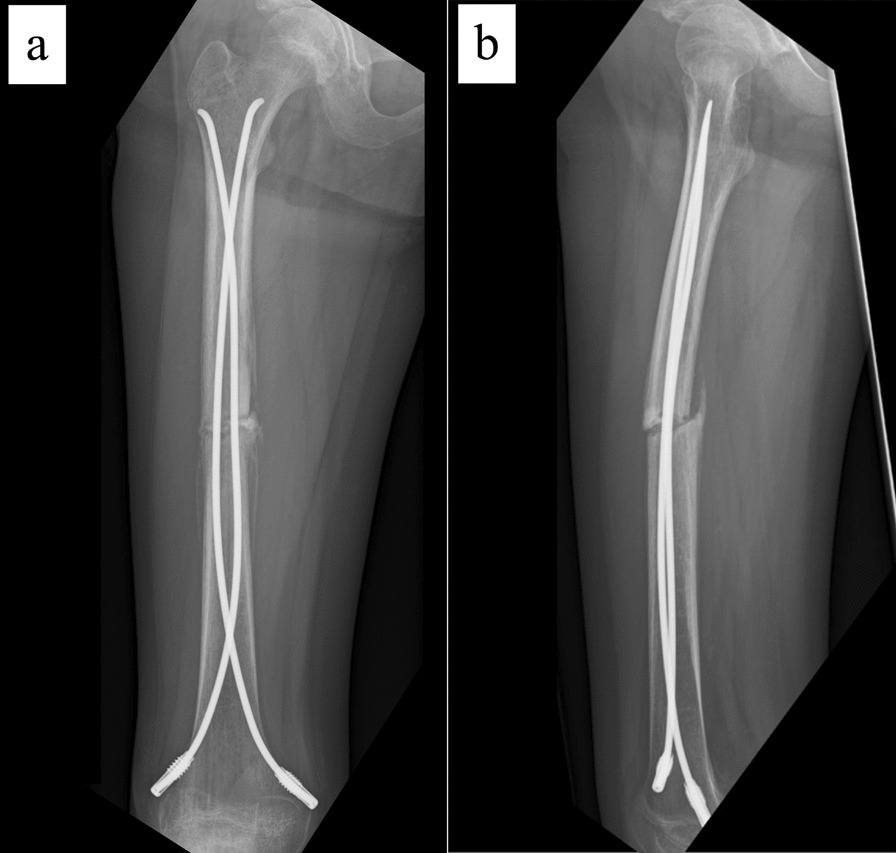
X-ray images at 5 months postoperatively. **a** Anteroposterior image and **b** lateral image. Delayed union is observed at the fracture site

**Fig. 5:**
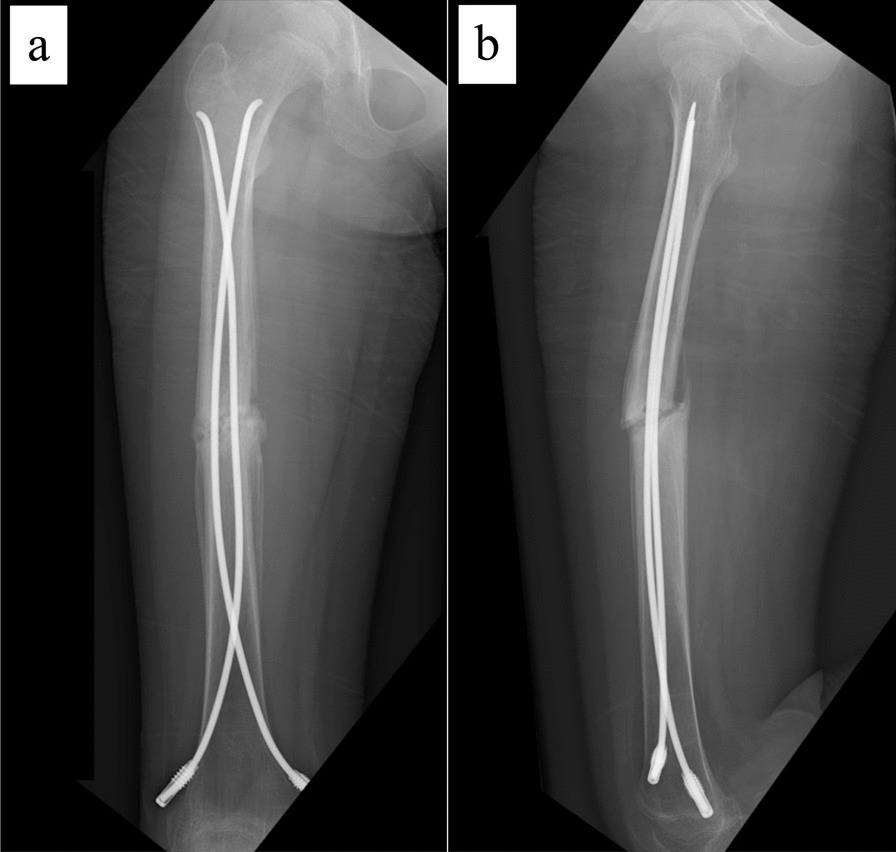
X-ray images at 6 months postoperatively. **a** Anteroposterior image and **b** lateral image. Nonunion is observed at the fracture site

**Fig. 6:**
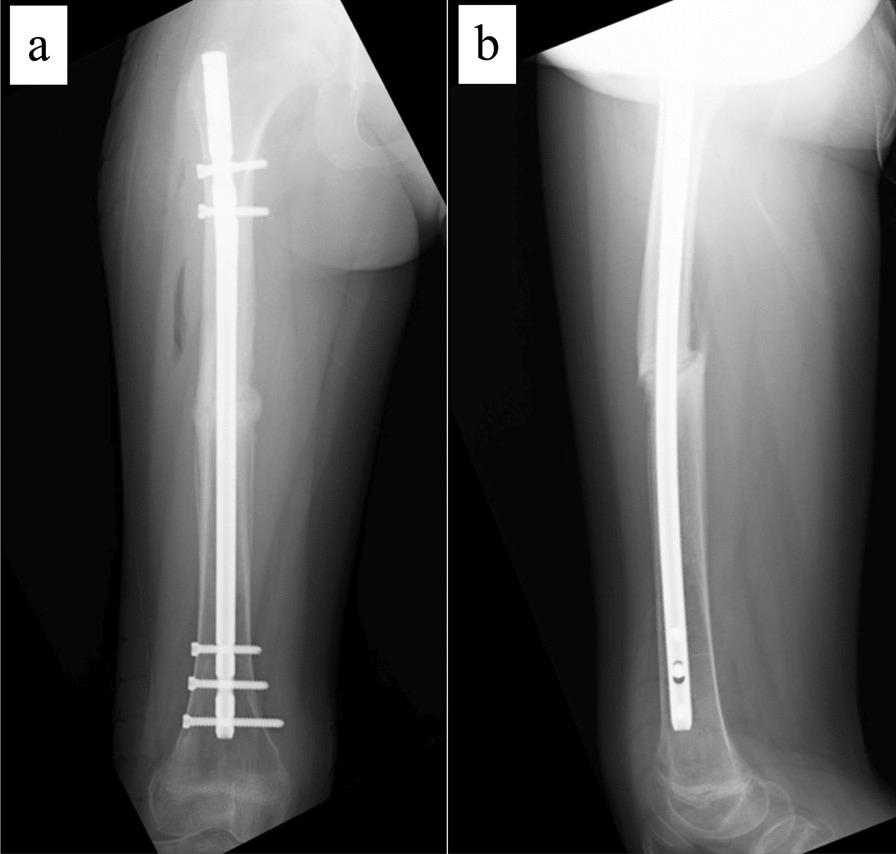
Postoperative radiographs of revision surgery. **a** Anteroposterior image and **b** lateral image. The two titanium elastic nails were removed and replaced with a rigid retrograde intramedullary nail. The fracture site was compressed, and the gap is reduced compared with preoperatively

**Fig. 7:**
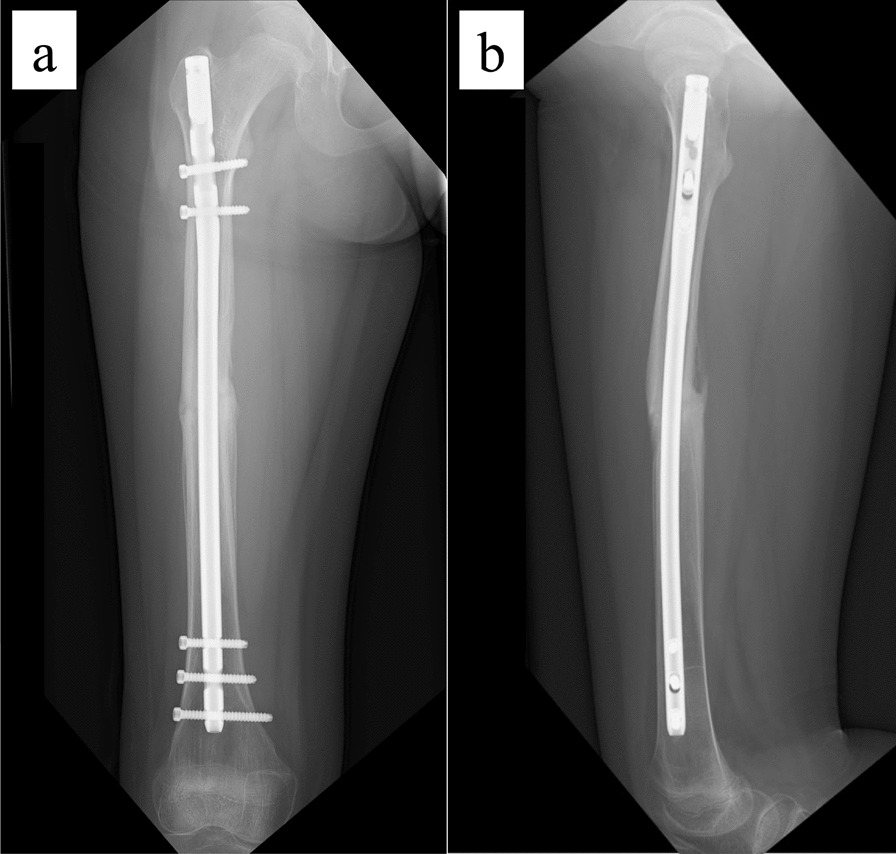
X-ray images and external views of the patient’s lower extremity 3 months after revision surgery. **a** Anteroposterior image and **b** lateral image. Bone fusion is observed in all images

**Fig. 8:**
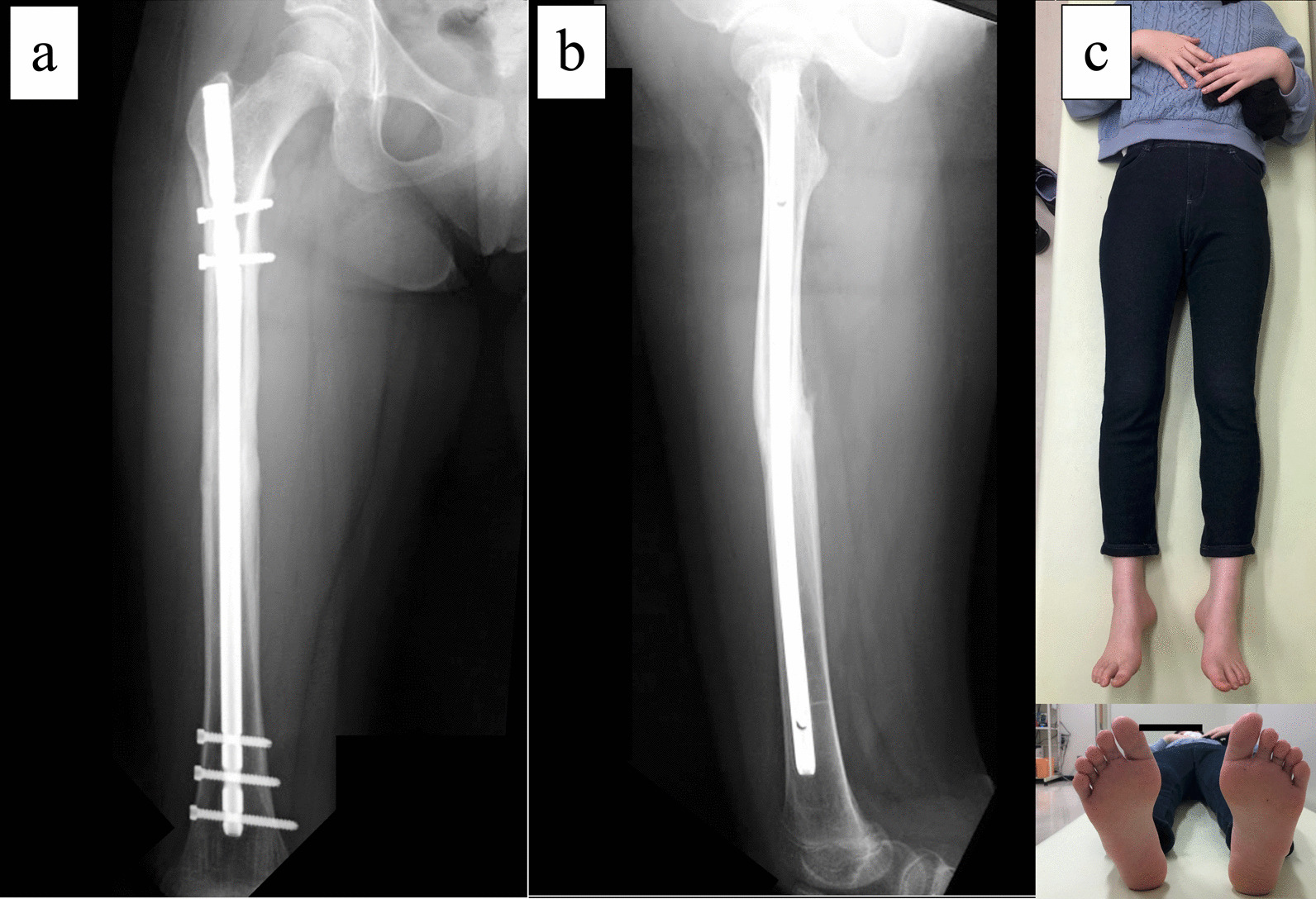
X-ray images and external views of the patient’s lower extremity 10 months after revision surgery. **a** Anteroposterior image, **b** lateral image, **c** photograph of both lower extremities, and **d** photograph of both feet viewed from the caudal side. There is no leg length discrepancy or rotational deformity

The patient is an 11-year-old, Japanese girl, with a weight of 38 kg and height of 147 cm (body mass index of 17.6 kg/m^2^). She was injured when she crashed into a wall after kickboarding down a hill and was brought to the emergency department, where radiographs showed a transverse fracture in the middle of the right femoral shaft with displacement (AO/OTA 32A3b) (Fig. 1). The patient also had an epiphyseal injury of the left olecranon, a fracture of the facial bone, and a dental injury, all of which were treated conservatively. She had no specific past or family history. Direct traction was performed after admission, and open osteosynthesis with retrograde insertion of two 3.5-mm-diameter titanium elastic nails (TEN, DePuy Synthes, Raynham, MA) was performed on day 7 after admission (Fig. 2). Stability was acceptable; therefore, no external bracing was required postoperatively. At 5 weeks postoperatively, callus was observed (Fig. 3), and partial weight-bearing was started, but low-intensity pulsed ultrasound (LIPUS) was also started due to concerns about delayed or absent callus formation. At 2 months postoperatively, the patient was able to walk with full weight bearing, but due to persistent pain and delayed fracture healing, a functional brace was placed on the right femur. At 3 months postoperatively, imaging showed delayed bone fusion (Fig. 4), and the patient was followed up with instructions for aggressive weight bearing and continued LIPUS. However, even at 6 months postoperatively, there was no tendency for fusion at the fracture site, with findings of atrophic or fibrotic nonunion (Fig. 5). Laboratory tests performed to determine the cause of the pseudoarthrosis showed vitamin D deficiency and vitamin K deficiency (Table 1). In addition, a pediatrician ruled out congenital endocrine or bone metabolic disorders. Thus, the decrease in biological activity due to vitamin D and K deficiencies was considered to be the main cause of the nonunion, and vitamin replacement therapy (15 mg of menatetrenone three times a day and 1 μg of alfacalcidol once a day) was started. In addition, to improve fracture site stability, the TEN was removed and replaced with a rigid antegrade intramedullary nail (T2 GTN, 10 mm in diameter and 320 mm long; Stryker, Kalamazoo, MI) 7 months after the initial surgery (Fig. 6). After the TEN was removed, there was mobility at the nonunion site. The entry point for the intramedullary nail was created slightly lateral to the greater trochanter, rather than medial or intra-articular. There was sclerotic scar tissue around the nonunion site, which was adequately reamed. After nail insertion, approximately 5 mm of compression was applied to the fracture site using a special device. Full weight-bearing was allowed postoperatively, and bone fusion was achieved 3 months after reoperation (Fig. 7). At 10 months postoperatively, no leg length discrepancy or rotational deformity was observed (Fig. 8).

**Table 1:** Results of biological parameters

AST	(U/L)	21	WBC	(× 10^3^/μL)	5.5
ALT	(U/L)	11	Neutrophil	(%)	58.0
LDH	(U/L)	163	Lymphocyte	(%)	27.0
ALP	(U/L)	392	Hb	(g/dL)	13.3
γ-GTP	(U/L)	16	PLT	(× 10^3^/μL)	250
CK	(U/L)	74			
TP	(g/dL)	7.4	Glucose	(mg/dL)	96
Albumin	(g/dL)	4.7	HbA1c	(%)	5.2
BUN	(mg/dL)	8.9			
Creatinine	(mg/dL)	0.38	I-PTH	(pg/mL)	57.6
UA	(mg/dL)	2.9	BALP	(U/L)	103
Sodium	(mmol/L)	143	TRACP-5b	(mU/dL)	> 1500
Potassium	(mmol/L)	4.9	Total P1NP	(μg/L)	751
Chlorine	(mmol/L)	108	ucOC	(ng/mL)	40.6
Calcium	(mg/dL)	9.7	25OHVD	(ng/mL)	< 4.0
IP	(mg/dL)	4.1			
CRP	(mg/dL)	< 0.03			

*AST* aspartate aminotransferase, *ALT* alanine aminotransferase, *LDH* lactate dehydrogenase, *ALP* alkaline phosphatase, *γ-GTP* γ-glutamyl transpeptidase, *CK* creatine kinase, *CK* total protein, *BUN* blood urea nitrogen, *UA* uric acid, *IP* inorganic phosphate, *CRP* C-reactive protein, *WBC* white blood cells, *Hb* hemoglobin, *PLT* platelet, *HbA1c* hemoglobin A1c, *I-PTH* intact-parathyroid hormone, *BALP* bone specific alkaline phosphatase, *TRACP-5b* tartrate-resistant acid phosphatase 5b, *Total P1NP* total procollagen type 1 N-terminal propeptide, *ucOC* undercarboxylated osteocalcin, *25OHVD* 25-hydroxy vitamin D

## Discussion and conclusion

The following two points were demonstrated in this case. Even children with no underlying disease may have vitamin deficiency, which may lead to decreased biological activity and postoperative nonunion in femoral shaft fractures, and rigid, antegrade intramedullary nails may be useful in the treatment of nonunion after femoral shaft fractures in children.

In children, obesity, lack of exposure to direct sunlight, and season (spring) have been reported as risk factors for vitamin D deficiency [5]; vitamin D deficiency is associated with increased risk and severity of fractures [6]. Vitamin K deficiency has also been found to be a risk factor for increased fractures in children, similar to vitamin D deficiency [7]. In addition, it has been suggested that vitamin D and K deficiencies not only increase fracture risk but also affect fracture healing. In adults, the baseline vitamin D status of patients with extremity fractures has been suggested to affect fracture healing [8]. A recent large cohort study showed that vitamin D deficiency is a risk factor (odds ratio: 2.15 and 95% confidence interval: 1.56–2.96) for nonunion of femoral shaft fractures [9]. Sprague *et al*. reported a beneficial effect of vitamin D supplementation in a systematic review of eight trials of the efficacy of vitamin D supplementation in fracture healing [10]. Several case reports have suggested that vitamin D deficiency may affect the formation of callus in fracture healing in children, and some have reported that vitamin D supplementation enhanced the formation of callus [11]. An animal study reported that vitamin D deficiency delayed fracture healing, and vitamin D supplementation increased callus formation [12]. In vitro studies have shown that vitamin D and vitamin K promote osteoblast differentiation at fracture sites [13]. However, the literature on the effects of vitamin supplementation on nonunion is very limited, and as far as we were able to ascertain, only case reports were available [14, 15].

In general, improving both biological activity and fixation of the fracture site is essential in the treatment of nonunion. However, there are reports of occult metabolic and endocrine abnormalities, including vitamin D deficiency, in many unexplained nonunions after osteosynthesis in adults. This suggests that, in the treatment of nonunion, attention is focused only on improving fixation, and surgical intervention is currently prioritized [16, 17].

In the present case, there was no history of possible causes of vitamin deficiency, such as lack of sun exposure or unbalanced diet, and the reason for the vitamin deficiencies was unknown. In addition, there was also no obvious failure of indication or technique in the initial surgery—it took 5 weeks for callus to appear, the nonunion was atrophic or fibrous, and bone fusion was achieved quickly after vitamin supplementation and reoperation. Therefore, the main reason for the nonunion in this case was considered to be the vitamin deficiencies. However, since this was a child with no specific comorbidity, no action was taken until the nonunion occurred. Once the delayed fracture healing was observed, a thorough evaluation for metabolic and endocrine abnormalities should have been performed.

Antegrade intramedullary nails may be useful in the treatment of nonunion after femoral shaft fractures in children. Complications of concern include epiphyseal injury, femoral head necrosis, and femoral overgrowth. It has been reported that femoral head necrosis is caused by damage to the circumflex femoral artery, and that nail insertion from the lateral aspect of the greater trochanter can avoid the risk of osteonecrosis [18, 19]. There are reports that it is not recommended for patients younger than 13 years old because of the high risk of epiphyseal injury; however, there are also reports of good outcomes in children older than 13 years old with no apparent complications [20, 21]. For femoral shaft nonunion after osteosynthesis without bone loss, conversion to closed intramedullary nailing has been reported to be effective, with the advantage of fewer complications and earlier weight bearing and rehabilitation. In addition, these reports indicate that chipping and bone grafting may be unnecessary in some cases, not only because of improved fixation, but also because reaming of the medullary cavity improves the biological activity of the fracture site [22–24].

In the present case, it cannot be completely ruled out that poor fixation in the first operation delayed fracture healing, and a second operation was necessary to ensure better fixation. Options for revision surgery included replacement of the TEN (φ4.0 mm), replacement with a rigid antegrade intramedullary nail, augmentation plating, and external fixation. In the present case, replacement with a rigid antegrade intramedullary nail that can be inserted from the lateral aspect of the greater trochanter was chosen because of the advantages of improved fixation, early postoperative weight bearing, and early recovery. The results, combined with the effects of vitamin supplementation, resulted in rapid callus formation and bone union. There are currently no apparent complications at 10 months postoperatively, but careful follow-up is still required. Considering the risks involved, we suggest that the use of progressive intramedullary nails in children should be limited to cases of nonunion or when other alternative methods are not indicated.

Even children with no underlying disease may have vitamin deficiency, which may lead to decreased biological activity and postoperative nonunion in femoral shaft fractures. In addition, rigid antegrade intramedullary nails may be useful in the treatment of nonunion after femoral shaft fractures in children. When delayed fracture healing occurs in children without an obvious specific comorbidity, as in the present case, a prompt search for abnormalities of bone metabolism and therapeutic intervention are necessary. Of particular importance is the possibility that cases of delayed union and nonunion associated with vitamin deficiency in children, such as the present case, may not be aggressively investigated and may be treated only with surgical approaches, such as revision surgery. Future studies should examine in detail the current status of vitamin deficiency in healthy children and its effect on fracture healing.

## Data Availability

Not applicable.

## Abbreviations

TEN: Titanium elastic nail
LIPUS: Low-intensity pulsed ultrasound
